# The learning curve of bilateral laparoscopic varicocelectomy: a prospective study

**DOI:** 10.1590/0100-6991e-20233456-en

**Published:** 2023-03-24

**Authors:** MIKHAEL BELKOVSKY, CARLO CAMARGO PASSEROTTI, LINDA FERREIRA MAXIMIANO, JOSÉ PINHATA OTOCH, JOSE ARNALDO SHIOMI DA CRUZ

**Affiliations:** 1 - Faculdade de Medicina da USP, Técnica Cirúrgica e Cirurgia Experimental - São Paulo - SP - Brasil; 2 - Hospital Alemão Oswaldo Cruz, Centro de Cirurgia Robótica - São Paulo - SP - Brasil

**Keywords:** Learning Curve, Urologic Surgical Procedures, Male, Varicocele, Curva de Aprendizado, Procedimentos Cirúrgicos Urológicos Masculinos, Varicocele

## Abstract

Varicocele occurs in 15% of general male population and in 35% of infertile men. Since 1992, surgical correction with laparoscopic varicocelectomy is the gold standard for treatment of symptomatic patients or patients with abnormal seminal analysis. The learning curve for this frequently performed procedure has not yet been described. In the present study, we investigated the learning curve of a single urologist in training performing his first 21 laparoscopic varicocelectomies using qualitative and quantitative tools to evaluate his performance during the process. Our results show that 14 bilateral laparoscopic varicocelectomies are enough to achieve the plateau of the learning curve.

## RESUMO

Varicocele ocorre em 15% dos homens e em 35% dos homens inférteis. Desde 1992, a correção cirúrgica com varicocelectomia laparoscópica é o padrão ouro para o tratamento de pacientes sintomáticos ou com análise seminal anormal. A curva de aprendizado para esse procedimento frequentemente realizado ainda não foi descrita. No presente estudo, investigamos a curva de aprendizado de um único urologista em treinamento realizando suas primeiras 21 varicocelectomias laparoscópicas usando ferramentas qualitativas e quantitativas para avaliar seu desempenho durante o processo. Nossos resultados mostram que 14 varicocelectomias laparoscópicas bilaterais são suficientes para atingir o platô da curva de aprendizado.

## INTRODUCTION

Varicocele is an abnormal dilatation of the pampiniform plexus, the plexus responsible for venous drainage and thermal regulation of the testes^1^. This condition is present in 15% of healthy men and in up to 35% of men with primary infertility. Despite being usually asymptomatic, varicocele can also present with scrotal discomfort, local edema, and other symptoms^2^ ^,^ ^3^.

Varicocele treatment is indicated in case of palpable varicocele, abnormality in seminal analysis, abnormal sperm function tests, or a 20% differential in testicular volume that is persistent for more than one year in adolescents. Varicocele correction can also be considered in patients with testicular pain or abnormal testosterone production^4^ ^,^ ^5^.

The first technique for varicocele treatment was open varicocelectomy. Currently, it has been used less frequently due to a higher rate of complications, with an incidence of 5 to 30%, including hydrocele, testicular atrophy, inadvertent ligation of the vas deferens, epididymitis, hematoma, and surgical site infections^6^.

Laparoscopic varicocelectomy was first introduced to treat male infertility in 1992 and almost all of its risks and benefits have been extensively studied^7^ ^,^ ^8^. More recently, new varicocelectomy techniques have been developed, such as subinguinal, microsurgical varicocelectomy, preferred in several guidelines for presenting slightly better results in relation to the reduction of complications and recovery of fertility^9^, robotic-assisted varicocelectomy^10^, laparoscopic varicocelectomy with lymphatic preservation^11^, and single-port varicocelectomy^12^. Even so, traditional laparoscopic varicocelectomy remains a viable alternative for varicocele correction^9^ ^,^ ^13^ ^,^ ^14^ .

The learning curve in surgery has become an increasingly frequent theme in the literature with the advent of robotic surgery, since unfamiliarity with the former would be an obstacle to the popularization of the latter. Thus, multiple studies have been published on robotic prostatectomy, nephrectomy, and pyeloplasty^15^ ^-^ ^19^. Following this trend, the learning curve of less complex procedures also began to be investigated, although to a lesser extent. The COVID-19 pandemic significantly increased the relevance of this topic, as the restrictions imposed by the pandemic drastically reduced the number of elective procedures available for training during residency programs^20^.

Consequently, the learning curve of the most diverse urological procedures began to be studied, such as correction of hypospadias^21^, implantation of penile prostheses^22^, urethroplasty^23^, percutaneous nephrolithotomy^24^, prostatic biopsy^25^, and others. Interestingly, we noticed a scarcity of studies on the learning curve of laparoscopic varicocelectomy.

The only study on the subject was published by Wang et al.^26^ and explored the differences in the varicocelectomy learning curve by comparing group A (who performed laparoscopic varicocelectomy with a laparoscopic training box) with group B (who trained with a virtual reality simulator). No statistical difference was observed between groups and the learning curve plateau was reached after 29 cases.

The primary objective of the present study is to describe, in an unprecedented way, the learning curve of bilateral laparoscopic varicocelectomy in supervised training, in adult patients^27^ ^-^ ^29^.

## METHODS

In this study, we analyzed the first 21 immediately consecutive bilateral laparoscopic varicocelectomies performed by the same surgeon. All patients had venous reflux present on preoperative Doppler and no longer had reflux on postoperative ultrasound performed after 6 months. The surgeries were divided into three groups according to the order performed: from the first to the seventh - Group I; from the eighth to the 14^th^ - Group II; and from the 15^th^ to the 21^st^ - Group III.

### Surgical procedure

**Figure 1:**
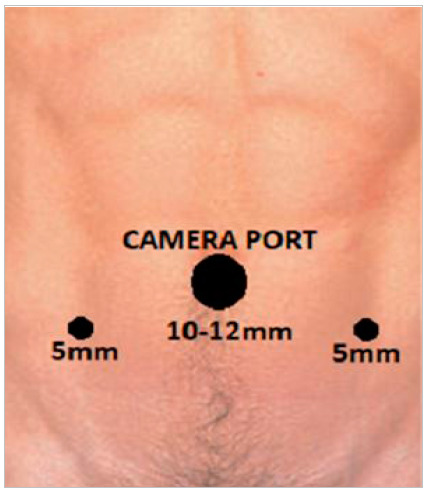
Trocar Positioning.

The patient, under general anesthesia, was placed in horizontal dorsal decubitus, slightly inclined in the Tredenlenburg position. A periumbilical incision was made with a Veress needle and pneumoperitoneum was inflated with a pressure of 10mmHg. A 10mm trocar was positioned in the umbilicus, and two other 5mm trocars were positioned approximately 8cm laterally to the camera trocar (Figure 1).

**Figure 2:**
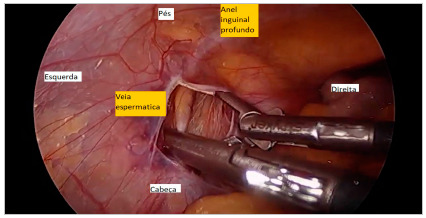
Inguinal ring dissection.

The parietal peritoneum was opened laterally to the iliac vessels, about 2cm from the deep inguinal ring, therefore far from the ureter (Figure 2). The right testicular vein was dissected, ligated with 3.0 cotton thread, and sectioned. The same procedure was performed contralaterally. Review of hemostasis was performed and removal of the trocars took place under direct vision.

### Data collection

We collected data regarding patients’ age, total operative time (in minutes), complications, and postoperative pain on the 1^st^ postoperative day, 7^th^ postoperative day, 1 month, and 6 months after the procedure, through a numerical scale from 1 to 10, 1 being described as no pain and 10 being the worst possible pain. In addition, we performed a qualitative analysis, based on a previously validated instrument^29^.

### Qualitative Score

To assess surgical skill, we used the GOALS (Global Assessment of Laparoscopic Skills) score, described by Vassiliou et al. in 2003^29^. GOALS was initially developed for the qualitative evaluation of laparoscopic surgeries by a human observer and has since been applied in several areas, from measuring the impact of using simulators for surgical learning to the development of machine learning models^30^ ^,^ ^31^. It considers 5 skill domains in laparoscopic surgery and assigns a score from 1 to 5 to each of them, according to Table 1.

**Table 1:** GOALS score.

Depth perception^a,b^
1 - Constantly overshoots target, wide swings, slow to correct
2.
3 - Some overshooting or missing target, but quick to correct
4.
5 - Accurately directs instruments in the correct plane to target
Bimanual dexterity^a,b^
1 - Uses only one hand, ignores non dominant hand, poor coordination between hands
2.
3 - Users both hands, but does not optimize interaction between hands
4.
5 - Expertly uses both hands in a complementary manner to provide optimal exposure
Efficiency^a,b^
1 - Uncertain, inefficient efforts; many tentative movements; constantly changing focus or persisting without progress
2.
3 - Slow, but planned movements are reasonably organized
4.
5 - Confident, efficient and safe conduct, maintains focus on task until it is better performed by way of an alternative approach
Tissue handling^a,b^
1 - Rough movements, tears tissue, injures adjacent structures, poor grasper control, grasper frequently slips
2.
3 - Handles tissue reasonably well, minor trauma to adjacent tissue (i.e., occasional unnecessary bleeding or slipping of the grasper)
4.
5 - Handles tissues well, applies appropriate traction, negligible injury to adjacent structures
Autonomy^a,b^
1. Unable to complete entire task, even with verbal guidance
2.
3. Able to complete task safely with moderate guidance
4.
5. Able to complete tasks independently without guidance.

^*a*^ *2= midterm between grades 1 and 3;* ^*b*^ *4 = midterm between degrees 3 and 5.*

### Statistical analysis

We evaluated the groups using the Kolmogorov-Smirnov test to confirm normal distribution. All variables showed normal distribution and were subsequently compared by ANOVA. Afterwards, we used the Tukey’s post-test for intergroup comparisons.

### Ethics and Financing

The study was approved by the Ethics Committee of the University Hospital of the Faculty of Medicine of USP and was conducted without funding sources.

## RESULTS

The age of the patients was similar between the studied groups: in group I, 30.01 years, with a Standard Deviation (SD) of ± 6.7; in group II, 33.5 years ± 3; in group III, 35.5 years ± 6.2.

The qualitative analysis of the GOALS variables showed improvement when comparing groups I and II in bimanual dexterity (p=0.007), depth perception (p=0.015), and autonomy (p=0.015). However, there was no difference when comparing groups II and III in any of the qualitative variables, as can be seen in Table 2.

**Table 2:** Qualitative analysis.

	Bimanual dexterity	Depth perception	Efficiency	Autonomy	Tissue handling
Group I (mean ± SD)	3±0	3±0.0	3.4±0.5	3±0	3.4±0.5
Group II (mean ± SD)	4±0.8	4.1±1.0	4.1±1.0	4.1±1.0	4.2±0.9
Group III (mean ± SD)	4.2±0.95	4.7±0.7	4.8±0.3	4.7±0.7	4.7±0.4
Group I vs II - p	0.007	0.015	0.14	0.015	0.06
Group II vs III - p	0.558	0.271	0.121	0.271	0.31

In the quantitative analysis, the total operative time (38.5±4.7 min vs 31.7±7.2 min, p=0.058) and pain on the 7^th^ postoperative day (3.5±1.5min vs. 1.8±1.4min, p=0.052) were significantly lower when comparing groups I and II. However, there was no difference between groups II and III regarding the observed quantitative variables (operative time, postoperative pain, and number of complications), as shown in Table 3.

**Table 3:** Quantitative analysis.

	Operative time (min)	Postoperative pain (Day 1)	Postoperative pain (Day 7)	Postoperative pain (1 month)	Postoperative pain (6 months)	Complications
Group I (mean ± SD)	38.5±4.7	4.8±2.0	3.5±1.5	0	0	0
Group II (mean ± SD)	31.7±7.2	3.4±1.3	1.8±1.4	0	0	0
Group III (mean ± SD)	31.7±2.8	3.8±0.9	1.8±1.4	0	0	0
Group I vs II - p	0.058	0.15	0.052	>0.999	>0.999	>0.999
Group II vs III - p	0.116	0.508	>0.999	>0.999	>0.999	>0.999

 We did not observe pain 1 month after the procedure, nor any postoperative complications (such as recurrence of varicocele, hydrocele, or testicular atrophy). In the control USG 6 months later, there was no clinically significant varicocele.

## DISCUSSION

The literature shows that laparoscopic repair presents better results than the open approach (shorter surgical time, less postoperative pain, shorter hospital stay, and earlier return to daily activities)^32^.

Scientific interest in understanding learning curves has grown dramatically over the past 20 years. Incidentally, the number of publications on the subject indexed in PubMed increased from 146 in 1996 to 1,070 in 2016. Research in the area takes place in different surgical procedures, such as hepatectomy, colorectal surgeries, and radical prostatectomy, with surgeons with different experiences, from beginners to seasoned ones^33^ ^,^ ^34^.

The description of the learning curve of bilateral laparoscopic varicocelectomy can be used as an essential tool for the development of more effective programs for surgical training, reducing the risk of complications and improving patients’ quality of life^35^.

In this study, we observed that laparoscopic varicocelectomy progresses with satisfactory clinical results from the first surgery. This is evidenced by non-recurrence of varicocele and absence of complications 1 and 6 months after the procedure.

Moreover, with professional training, there is also a statistically significant improvement in surgical time, surgical skill measured by the qualitative score, and postoperative pain in the first week. The absence of statistical difference in these variables between groups II and III allows us to infer that the plateau of the laparoscopic varicocelectomy learning curve occurs after 14 surgeries.

The reduction in surgical time observed in the present study, concomitant with the increase in surgical skill as the surgeon accumulates experience, are in line with previous results demonstrated in the literature^36^ ^,^ ^37^. Reduction in operative time reduces anesthetic time and the probability of postoperative complications^32^.

Wang et al.^26^ observed the learning curve plateau with 29 procedures, but did not observe whether there is a correlation between the learning curve in the simulator and in practice. Our study, however, featured a faster plateau curve directly in the patient, which raises the question of the place of simulators in learning laparoscopic varicocelectomy. Perhaps because it is a technically simple procedure, a surgeon familiar with laparoscopy can easily master the technique.

The external validity of the results is limited because we used a retrospective analysis of a database prospectively fed with data from a single surgeon. Even so, considering the difficulty of accessing data on the surgical learning curve and ethical issues related to patient safety and confidentiality, it is still customary for such studies to describe the learning curve of a single surgeon, especially for urologists^16^ ^-^ ^18^. In addition, we did not assess anatomical variations or differences in BMI between patients, factors that could interfere with learning difficulties, and the comparative seminal analysis was not available for statistical analysis.

On the other hand, surgical learning in patients with varicocele is a point that increases the reliability of the learning curve when compared to artificial or in vitro models. Furthermore, this study dispensed with special, high-cost disposable materials (such as robotic and microscopic materials, ultrasonic scalpels, and disposable clips), using only permanent instruments, which makes it more faithful to the Brazilian reality^4^ ^,^ ^7^ ^,^ ^38^.

## CONCLUSION

We did not observe statistical difference in the studied parameters (surgical skill and total operative time) between groups II and III. Thus, we can estimate that 14 bilateral laparoscopic varicocelectomies are enough for a surgeon to reach proficiency in the learning curve.

## References

[1] Masson P, Brannigan RE (2014). The varicocele. Urol Clin North Am.

[2] Chiba K, Ramasamy R, Lamb D, Lipshultz L (2016). The varicocele diagnostic dilemmas, therapeutic challenges and future perspectives. Asian J Androl.

[3] Alsaikhan B, Alrabeeah K, Delouya G, Zini A (2016). Epidemiology of varicocele. Asian J Androl.

[4] Kwak N, Siegel D (2014). Imaging and interventional therapy for varicoceles. Curr Urol Rep.

[5] Whelan P, Levine L (2016). Effects of varicocelectomy on serum testosterone. Transl Androl Urol.

[6] Chan P (2011). Management options of varicoceles. Indian J Urol.

[7] Hagood PG, Mehan DJ, Worischeck JH, Andrus CH, Parra RO (1992). Laparoscopic varicocelectomy preliminary report of a new technique. J Urol.

[8] Ding H, Tian J, Du W, Zhang L, Wang H, Wang Z (2012). Open non-microsurgical, laparoscopic or open microsurgical varicocelectomy for male infertility a meta-analysis of randomized controlled trials. BJU Int.

[9] Shah R, Agarwal A, Kavoussi P, Rambhatla A, Saleh R, Cannarella R (2023). Consensus and Diversity in the Management of Varicocele for Male Infertility Results of a Global Practice Survey and Comparison with Guidelines and Recommendations. World J Mens Health.

[10] McCullough A, Elebyjian L, Ellen J, Mechlin C (2018). A retrospective review of single-institution outcomes with robotic-assisted microsurgical varicocelectomy. Asian J Androl.

[11] Liang Z, Guo J, Zhang H, Yang C, Pu J, Mei H (2011). Lymphatic Sparing Versus Lymphatic Non-Sparing Laparoscopic Varicocelectomy in Children and Adolescents A Systematic Review and Meta-Analysis. Eur J Pediatr Surg.

[12] Kaouk JH, Palmer JS (2008). Single-port laparoscopic surgery initial experience in children for varicocelectomy. BJU Int.

[13] Bharathidasan R, Jayaprakash R, Bhaskar S, Ambujam G (2017). Laparoscopic varicocelectomy now the gold standard procedure for varicocele - A comparative study with open technique based on our experience. IAIM,.

[14] Wang H, Ji ZG (2020). Microsurgery Versus Laparoscopic Surgery for Varicocele A Meta-Analysis and Systematic Review of Randomized Controlled Trials. J Invest Surg.

[15] Saito FJA, Dall'Oglio MF, Ebaid GX, Bruschini H, Chade DC, Srougi M (2011). Learning curve for radical retropubic prostatectomy. Int Braz J Urol.

[16] Ploussard G, Salomon L, Parier B, Abbou CC, de la Taille A (2013). Extraperitoneal robot-assisted laparoscopic radical prostatectomy a single-center experience beyond the learning curve. World J Urol.

[17] Ou YC, Yang CR, Wang J, Yang CK, Cheng CL, Patel VR (2011). The learning curve for reducing complications of robotic-assisted laparoscopic radical prostatectomy by a single surgeon. BJU Int.

[18] Polok M, Dzielendziak A, Apoznanski W, Patkowski D (2019). Laparoscopic Heminephrectomy for Duplex Kidney in Children-The Learning Curve. Front Pediatr.

[19] Tasian GE, Wiebe DJ, Casale P (2013). Learning Curve of Robotic Assisted Pyeloplasty for Pediatric Urology Fellows. J Urol.

[20] Porpiglia F, Checcucci E, Amparore D, Verri P, Campi R, Claps F (2020). Slowdown of urology residents' learning curve during the COVID-19 emergency. BJU Int.

[21] Hisamatsu E, Sugita Y, Haruna A, Shibata R, Yoshino K (2021). The learning curve in proximal hypospadias repair. J Pediatr Urol.

[22] Barrier A, Marcelli F, Villers A (2019). Courbe d'apprentissage d'implantation de prothèse pénienne. Prog Urol.

[23] Choi J, Lee CU, Sung HH (2020). Learning curve of various type of male urethroplasty. Investig Clin Urol.

[24] Sahan M, Sarilar O, Savun M, Caglar U, Erbin A, Ozgor F (2020). Adopting for Supine Percutaneous Nephrolithotomy Analyzing the Learning Curve of Tertiary Academic Center Urology Team. Urology.

[25] Checcucci E, Piramide F, Amparore D, de Cillis S, Granato S, Sica M (2021). Beyond the Learning Curve of Prostate MRI/TRUS Target Fusion Biopsy after More than 1000 Procedures. Urology.

[26] Wang Z, Ni Y, Zhang Y, Jin X, Xia Q, Wang H (2014). Laparoscopic varicocelectomy virtual reality training and learning curve. JSLS.

[27] Sandy NS, Cruz da JAS, Passerotti CC, Nguyen H, Reis dos ST, Gouveia EM (2013). Can the learning of laparoscopic skills be quantified by the measurements of skill parameters performed in a virtual reality simulator. Int Braz J Urol.

[28] da Cruz JAS, dos Reis ST, Cunha Frati RM, Duarte RJ, Nguyen H, Srougi M (2016). Does Warm-Up Training in a Virtual Reality Simulator Improve Surgical Performance A Prospective Randomized Analysis. J Surg Educ.

[29] Vassiliou MC, Feldman LS, Andrew CG, Bergman S, Leffondré K, Stanbridge D (2005). A global assessment tool for evaluation of intraoperative laparoscopic skills. Am J Surg.

[30] Esteva A, Chou K, Yeung S, Naik N, Madani A, Mottaghi A (2021). Deep learning-enabled medical computer vision. NPJ Digit Med.

[31] Sroka G, Feldman LS, Vassiliou MC, Kaneva PA, Fayez R, Fried GM (2010). Fundamentals of Laparoscopic Surgery simulator training to proficiency improves laparoscopic performance in the operating room-a randomized controlled trial. Am J Surg.

[32] Cheng H, Clymer JW, Po-Han Chen B, Sadeghirad B, Ferko NC, Cameron CG (2018). Prolonged operative duration is associated with complications a systematic review and meta-analysis. J Surg Res.

[33] Kluger MD, Vigano L, Barroso R, Cherqui D (2013). The learning curve in laparoscopic major liver resection. J Hepatobiliary Pancreat Sci.

[34] da Cruz JAS, Passerotti CC, Frati RMC, Reis dos ST, Okano MTR, Gouveia ÉM (2012). Surgical performance during laparoscopic radical nephrectomy is improved with training in a porcine model. J Endourol.

[35] Nandyala S, Fineberg SJ, Pelton M, Singh K (2014). Minimally invasive transforaminal lumbar interbody fusion one surgeon's learning curve. Spine J.

[36] Shetye KR, Kavoussi LR, Ramakumar S, Fugita OE, Jarrett TW (2003). Laparoscopic renal biopsy a 9-year experience. BJU Int.

[37] Guo J, Zeng Z, Cao R, Hu J (2019). Intraoperative serious complications of laparoscopic urological surgeries a single institute experience of 4,380 procedures. Int Braz J Urol.

[38] Micali S, Ghaith A, Martorana E, Zordani A, Territo A, Bianchi G (2014). Bilateral spermatic cord en bloc ligation by laparoendoscopic single-site surgery preliminary experience compared to conventional laparoscopy. BMC Urol.

